# Combination of body mass index and albumin predicts the survival in metastatic castration‐resistant prostate cancer patients treated with abiraterone: A post hoc analysis of two randomized trials

**DOI:** 10.1002/cam4.4205

**Published:** 2021-08-20

**Authors:** Jian Pan, Jun Wang, Yu Wei, Tingwei Zhang, Sheng Zhang, Dingwei Ye, Yao Zhu

**Affiliations:** ^1^ Department of Urology Fudan University Shanghai Cancer Center Shanghai China; ^2^ Department of Oncology Shanghai Medical College Fudan University Shanghai China; ^3^ State Key Laboratory of Oncology in South China Collaborative Innovation Center for Cancer Medicine Sun Yat‐sen University Cancer Center Guangzhou China; ^4^ Department of Urology Sun Yat‐sen University Cancer Center Guangzhou China; ^5^ Department of Medical Oncology Fudan University Shanghai Cancer Center Shanghai China

**Keywords:** body mass index, metastatic castration‐resistant prostate cancer, serum albumin, survival

## Abstract

**Background:**

Low body mass index (BMI) and low serum albumin levels are suggested indicators of malnutrition and are associated with poor outcomes in cancer patients. Decreasing androgen can alter lipid metabolism, so the prognostic value of BMI may change in metastatic castration‐resistant prostate cancer (mCRPC) patients receiving abiraterone. We aimed to delineate the prognostic value of BMI, serum albumin, and BMI and serum albumin (ALB) combined.

**Materials and methods:**

A post hoc analysis was performed on data from two randomized clinical trials evaluating the efficacy of abiraterone in chemotherapy‐pretreated and ‐naïve mCRPC patients. Survival analysis was conducted using Kaplan–Meier and Cox proportional hazard methods.

**Results:**

A total of 2,205 mCRPC patients were included in this study. Low ALB independently predicted the OS in both cohorts (HR, 1.54; 95%CI, 1.34–1.78 and HR, 1.40; 95%CI, 1.21–1.64, respectively), while low BMI independently predicted the OS only in the post‐chemotherapy cohort (HR, 1.30; 95%CI, 1.12–1.50) but not in the pre‐chemotherapy cohort (HR, 1.19; 95%CI, 0.98–1.43). By combining BMI (<25 kg/m^2^ or ≥30 kg/m^2^) and ALB (<4 g/dl or >4 g/dl), the four groups were characterized and their HRs were 1, 0.60 (95%CI, 0.47–0.76, *p *< 0.001), 0.75 (95%CI,0.61–0.92 *p* = 0.006), and 0.49 (95%CI, 0.41–0.60, *p *< 0.001) in post‐chemotherapy patients and 1, 0.64 (95%CI, 0.46–0.89, *p* = 0.008), 0.75 (95%CI,0.58–0.98 *p* = 0.034), and 0.55 (95%CI, 0.42–0.72, *p *< 0.001) in chemotherapy‐naïve patients, respectively.

**Conclusions:**

Our results demonstrate that the combination of BMI and ALB better characterizes the risk groups irrespective of previous chemotherapy. Patients with high BMI but low ALB have higher risk of death than patients with low BMI but high ALB.

## Graphical abstract

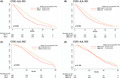

**Take home message**

Combination of BMI and ALB better characterizes the risk groups irrespective of previous chemotherapy. Patients with high BMI but low ALB have higher risk of death than patients with low BMI but high ALB.

## 1 INTRODUCTION

Identification of patients with malignancy at high risk of poor outcome and poor therapeutic response is vital for treatment decision‐making. Body mass index (BMI) has been widely evaluated for its association with tumor progression, clinical outcome, and treatment response, especially to targeted and immune therapies.^1^, ^2^ Previous studies reported that overweight (BMI 25.0–29.9 kg/m^2^) and obesity (BMI ≥30 kg/m^2^) are associated with increased risk of many cancer types,^3^, ^4^ but obese patients with melanoma have better survival benefit from targeted or immune therapies.^1^, ^2^ The prevalence of overweight and obesity has been increasing worldwide, with more than two thirds of adults in the United States being overweight or obese in 2013–2014.^5^, ^6^ However, evidence of an association of overweight and obesity with cancer outcome is insufficient and remains to be further elucidated.

Serum albumin (ALB) is commonly used as an indicator of nutritional status and reflects the degree of malnutrition. Low serum ALB has been widely used as a prognostic factor associated with poor outcome in cancer^7^, ^8^ and co‐occurrence with low BMI (BMI < 20 kg/m^2^) is considered to be cachexia.^9^ In addition, ALB is also an easy‐to‐use and highly predictive tool for the assessment of inflammation in cancer patients^10^ while systemic inflammation predicts muscle mass wasting in progressive cancer. Patients with advanced cancer, for example, metastatic castration‐resistant prostate cancer (mCRPC), tend to receive multiple lines of treatment. The incidence of cancer‐related or treatment‐induced malnutrition is increased in this population and may lead to limited drug exposure. Therefore, early identification of patients with potential malnutrition is of great importance to improve the quality of life and prognosis of patients with advanced cancer.

To our knowledge, this is the first study to examine the impact of BMI, ALB, and their combined effect on the outcome of mCRPC patients treated with next‐generation AR‐directed therapy (abiraterone) before or after chemotherapy. We first examined the association of BMI and ALB with cancer survival in 2,205 mCRPC patients. Subsequently, we combined BMI and ALB to evaluate their impact on survival. We hypothesized BMI and ALB are independently associated with cancer outcome and that combining both risk factors may better identify patients at higher risk of malnutrition and poor outcome.

## 2 MATERIALS AND METHODS

### 2.1 Patient population

COU‐AA‐301 and COU‐AA‐302 were phase III, multicenter, randomized, double‐blind, placebo‐controlled studies evaluating the efficacy and safety of 1,000 mg daily abiraterone acetate (AA) plus 5 mg twice‐daily prednisone (abiraterone arm) versus placebo plus prednisone (prednisone arm) in chemotherapy‐pretreated and ‐naïve patients with mCRPC.

Both study designs have been previously reported in detail.^11^, ^12^ In COU‐AA‐301, patients who had received docetaxel chemotherapy over a maximum of two cycles and had disease progression with a serum testosterone concentration lower than 50 ng/dL were included. In COU‐AA‐302, the study enrolled patients with chemotherapy‐naive mCRPC who were medically or surgically castrated, had tumor progression, and were asymptomatic or mildly symptomatic, as defined by the Brief Pain Inventory‐Short Form (asymptomatic with scores of 0 or 1 or mildly asymptomatic with scores of 2–3) while patients with visceral metastases or patients who had received previous therapy with ketoconazole for >7 days were excluded. In addition, in COU‐AA‐301, 1,195 patients were randomized (2:1) into the abiraterone and prednisone arms while in COU‐AA‐302, 1,088 patients were randomized (1:1).

Our analysis utilized the final dataset from COU‐AA‐301 and COU‐AA‐302. We included patients from both studies except those not receiving study drugs according to the study protocol or whose BMI or ALB information was not available. This study, carried out under YODA Project 2017–1356, used data obtained from the Yale University Open Data Access Project, which has an agreement with Janssen Research & Development, LLC. Our study was approved by the Medical Ethics Committee of Fudan University Shanghai Cancer Center prior to conducting the study.

## 3 COVARIATE ASSESSMENT AND OUTCOME MEASURES

Clinical information on height, weight, health characteristics (ECOG and BPI‐SF), laboratory examinations that performed at the time of enrollment (PSA, Hb, LDH, ALP, and ALB), and previous treatments (chemotherapy regimens) were retrieved from the YODA project. BMI was calculated using the first clinical visit data for height and weight. The end point was overall survival (OS). OS was defined as time from randomization to death from any cause.

### 3.1 Statistical analysis

Baseline characteristics were calculated by descriptive statistics (mean [SD] and percentages). Kaplan–Meier survival curves and log‐rank tests were used to evaluate the OS by categories of BMI (<25 kg/m^2^ or ≥30 kg/m^2^), ALB (<4 g/dl or ≥4 g/dl), and combined BMI and ALB. Multivariable adjusted hazard ratios (HRs) and corresponding 95% CIs for OS were estimated using Cox proportional hazards model. Covariates included in the multivariate Cox model were BMI, ALB, or BMI and ALB combined, treatment (abiraterone acetate + prednisone vs. placebo + prednisone), ECOG score, previous chemotherapy regimens (only in COU‐AA‐301), progression category (only in COU‐AA‐301), and PSA. All statistical analyses were performed using STAT version 9.3 (StataCorp LLC). Statistical significance was established with two‐sided tests with *α* = 0.05.

## 4 RESULTS

### 4.1 Characteristics of the study cohort

Of 2,205 mCRPC patients, 1,172 were previously treated with docetaxel and 1,033 were chemotherapy‐naïve. The proportion of patients with excess body weight (≥25 kg/m^2^) was higher in the post‐chemotherapy cohort (n = 809, 69.0%) than in the pre‐chemotherapy cohort (n = 858, 82%). Detailed baseline characteristics of all patients are listed in Table S1. By combining BMI and ALB, patients were divided into four groups (group 1: BMI<25, ALB≤4; group 2: BMI<25, ALB>4; group 3: BMI≥25, ALB≤4; and group 4: BMI≥25, ALB>4). The proportion of these groups was 15.5%, 15.4%, 27.6%, and 40.2% in the post‐chemotherapy cohort and 8.7%, 9.6%, 42.6%, and 39.1% in the pre‐chemotherapy cohort, respectively. High BMI but low ALB patients represented the major proportion of the cohorts, especially in the pre‐chemotherapy cohort. Detailed baseline characteristics of these four groups are listed in Table 1.

**TABLE 1:** Baseline characteristics of patients in the (A) COU‐AA‐301 cohort (B) COU‐AA‐301 cohort

(A)				
	COU‐AA−301 (n = 1172)			
	Group 1 (BMI < 25, ALB≤4, n = 182)	Group 2 (BMI < 25, ALB>4, n = 181)	Group 3 (BMI≥25, ALB≤4, n = 324)	Group 4 (BMI≥25, ALB>4, n = 471)
ECOG, N (%)				
1	144	164	275	421
2	31	17	38	36
BPI‐SF, N (%)				
0	30	41	73	96
1	31	44	63	117
2	54	37	82	121
3	43	38	63	99
4	14	12	22	21
Previous chemotherapy regimens, N (%)				
1	108	121	213	344
2	66	56	99	118
Progression category, N (%)				
PSA only	42	61	86	148
Radiographic	132	116	229	314
Median PSA, ng/ml (IQR)	237.6 (66.0–683.4)	111.8 (42.7–409.3)	127.7 (37.4–387.8)	95.8 (31.5–306.2)
Median Hb, g/dl (IQR)	10.8 (10.0–11.9)	12.15 (11–13.2)	11.4 (10.2–12.5)	12.4 (11.5–13.2)
Median LDH, IU/l (IQR)	269 (187.5–402.5)	226 (187.2–324.8)	233.5 (187–318.5)	221 (186.2–288)
Median ALP, IU/l (IQR)	189.5 (97.5–353.2)	117 (77–295)	136.5 (83.5–264.2)	108 (75.3–214.5)

(B)				
	COU‐AA−302 (n = 1033)			
	Group 1 (BMI < 25, ALB≤4, n = 90)	Group 2 (BMI < 25, ALB>4, n = 99)	Group 3 (BMI≥25, ALB≤4, n = 440)	Group 4 (BMI≥25, ALB>4, n = 404)
ECOG, N (%)				
0	62	77	306	322
1	28	22	126	77
2			1	
BPI‐SF, N (%)				
0	39	52	213	211
1	11	13	56	68
2	19	18	79	50
3	18	14	74	61
4	1	1		1
Median PSA, ng/ml (IQR)	50.7 (17.2–141.8)	34.9 (12.8–97.5)	42.4 (17.1–113.6)	30.6 (11.6–79.6)
Median Hb, g/dl (IQR)	12.6 (11.8–13.4)	13.3 (12.6–14.0)	12.9 (12.1–13.7)	13.6 (12.8–14.2)
Median LDH, IU/l (IQR)	194.0 (172.5–233.5)	180.0(159.0–212.0)	183.0 (158.5–215.5)	187 (167.8–212.2)
Median ALP, IU/l (IQR)	114.5 (82.8–186.5)	77 (61–118.5)	92 (72–132.5)	85 (70–118.5)

Abbreviations: AAP, abiraterone acetate +prednisone; ALB, albumin; ALP, alkaline phosphatase; BMI, body mass index; BPI‐SF, Brief Pain Inventory‐Short Form; ECOG, Eastern Cooperative Oncology Group; Hb, hemoglobin; IQR, interquartile range; LDH, lactate dehydrogenase;PP, placebo +prednisone; PSA, prostate‐specific antigen.

## 5 ASSOCIATION OF BMI AND CANCER SURVIVAL

**FIGURE 1:**
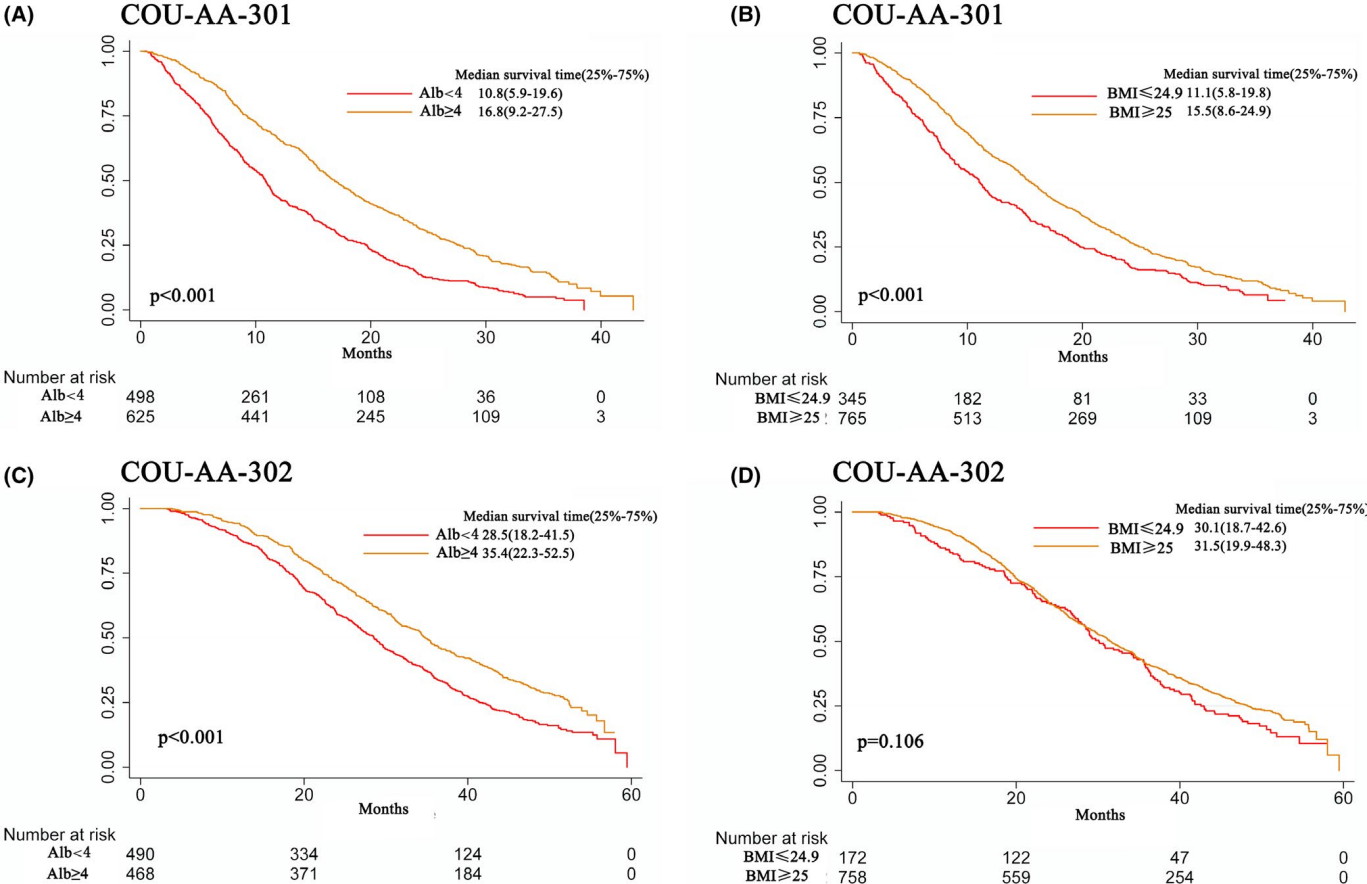
Kaplan–Meier showing the survival of patients in COU‐AA‐301 stratified by albumin (A) and BMI (B) and patients in COU‐AA‐302 stratified by albumin (C) and BMI (D)

As shown in Figure 1B, post‐chemotherapy patients with normal BMI (BMI < 25 kg/m^2^) had poorer OS than patients with a high BMI, whereas pre‐chemotherapy patients had no significant survival differences regardless of BMI (Figure 1D) (log‐rank, *p *< 0.001). In multivariable analyses, after adjustment by treatment, ECOG, previous chemotherapy regimens, progression category, PSA, and BMI independently predicted the OS only in the post‐chemotherapy cohort (HR, 1.30; 95%CI, 1.12–1.50) but not in the pre‐chemotherapy cohort (HR, 1.19; 95%CI, 0.98–1.43).

### 5.1 Association of serum albumin and cancer survival

Kaplan–Meier curves demonstrated that patients with low ALB (Figure A and C), had worse OS than those with high ALB both in the pre‐ and post‐chemotherapy cohorts (log‐rank, *p *< 0.001). In multivariate analyses, serum albumin independently predicted the OS both in COU‐AA‐301 and COU‐AA‐302 (HR, 1.54; 95%CI, 1.34–1.78 and HR, 1.40; 95%CI, 1.21–1.64, respectively; all *p* values < 0.001).

### 5.2 Combined effect of BMI and serum albumin on cancer survival

**FIGURE 2:**
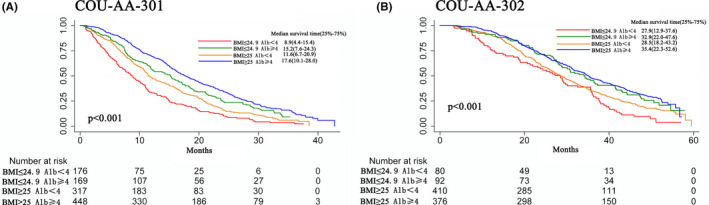
Kaplan–Meier showing the survival of patients in COU‐AA‐301 (A) and COU‐AA‐302 (B) stratified by combing BMI and albumin

As observed in the Kaplan–Meier curves (Figure 2), in both cohorts, patients with normal BMI and low ALB had the worst survival, whereas patients with high BMI and high ALB survived the longest (log‐rank, *p *< 0.001). Interestingly, patients with high BMI but low ALB had poorer survival than patients with normal BMI but high ALB. In multivariate analyses, the HRs of low BMI combined with low ALB, low BMI combined with high ALB, and high BMI combined with low ALB for OS were 1, 0.60 (95%CI, 0.47–0.76; *p *< 0.001), 0.75 (95%CI,0.61–0.92; *p* = 0.006), and 0.49 (95%CI, 0.41–0.60; *p *< 0.001) in post‐chemotherapy patients and 1, 0.64 (95%CI, 0.46–0.89; *p* = 0.008), 0.75 (95%CI, 0.58–0.98; *p* = 0.034), and 0.55 (95%CI, 0.42–0.72; *p *< 0.001), respectively (Table 2).

**TABLE 2:** Cox regression analysis for the end point overall survival in the COU‐AA‐301 and COU‐AA‐302 cohorts

	COU‐AA−301		COU‐AA−302	
	HR (95% CI)	*p* value	HR (95% CI)	*p* value
Univariate Cox model				
BMI (<25kg/m^2^ vs. ≥25kg/m^2^)	1.38 (1.21–1.59)	<0.001	1.16 (0.97–1.40)	0.107
ALB (≤4g/dl vs. >4g/dl)	1.69 (1.49–1.92)	<0.001	1.49 (1.28–1.72)	<0.001
Group				
1 (ref.)	—	—	—	—
2	0.55 (0.44–0.69)	<0.001	0.60 (0.43–0.83)	0.002
3	0.71 (0.59–0.86)	0.001	0.74 (0.58–0.96)	0.023
4	0.45 (0.37–0.54)	<0.001	0.52 (0.40–0.67)	<0.001
Multivariate Cox model^a^				
BMI (<25kg/m^2^ vs. ≥25kg/m^2^)	1.30 (1.12–1.50)	<0.001	1.19 (0.98–1.43)	0.076
ALB (≤4g/dl vs. >4g/dl)	1.54 (1.34–1.78)	<0.001	1.40 (1.21–1.64)	<0.001
Group				
1 (ref.)	—	—	—	—
2 (ref.)	0.60 (0.47–0.76)	<0.001	0.64 (0.46–0.89)	0.008
3 (ref.)	0.75 (0.61–0.92)	0.006	0.75 (0.58–0.98)	0.034
4 (ref.)	0.49 (0.41–0.60)	<0.001	0.55 (0.42–0.72)	<0.001

Abbreviations: BMI, body mass index; ECOG, Eastern Cooperative Oncology Group; HR, hazard ratio; PSA, prostate‐specific antigen.

^a^ Adjusted for treatment (abiraterone acetate + prednisone vs. placebo + prednisone), ECOG score, previous chemotherapy regimens, progression category, and PSA.

## 6 DISCUSSION

In this post hoc analysis of two large independent cohorts of patients with mCRPC treated with abiraterone acetate or placebo, we found the combination of BMI and ALB was strongly associated with OS, suggesting that these easily acquired clinical data would be useful in identifying patients at higher risk of poor outcome. Patients with normal BMI but low ALB had a remarkable risk of death in both first‐line and second‐line abiraterone. Furthermore, we identified an under‐recognized highly prevalent group of patients with high BMI but low ALB who had inferior clinical outcomes than conventional low BMI but normal ALB patients. Nearly one fifth and two fifths of patients in these two clinical trials with strict inclusion and exclusion criteria indicated an even higher proportion of unrevealed high BMI patients at high risk of poor survival in the real world. Our findings reinforce the idea that hypoalbuminemia high BMI, an overlooked subgroup, is prominent after first‐line treatment of mCRPC and warrants careful consideration.

Obesity is a strong risk factor for the incidence of aggressive prostate cancer. However, its association with survival, especially in advanced prostate cancer, is still controversial.^13^, ^14^ Data from Cancer and Leukemia Group B, which included nine prospective clinical trials and totaled 1,296 CRPC patients diagnosed between 1991 and 2004 suggested that higher BMI (≥25 kg/m^2^) was associated with longer OS and lower risk of prostate cancer‐specific mortality.^15^ Similar studies on CRPC patients treated with docetaxel supported this conclusion.^16^, ^17^ However, a phase III clinical study of docetaxel versus mitoxantrone, TA327, which involved 1,006 mCRPC patients, showed that BMI was not correlated with OS.^18^ It should be noted that the above studies included patients mainly treated with chemotherapy and that the treatment regimens for mCRPC have been changed since the approval of next‐generation anti‐androgen drugs (e.g., abiraterone acetate and enzalutamide). Therefore, the association of BMI with survival in patients treated with next‐generation anti‐androgen drugs remains poorly understood. Therefore, we conducted this post hoc study, the largest to date to our knowledge, to examine the relationship between BMI and survival in mCRPC patients treated with abiraterone, and found BMI was associated with survival only in post‐chemotherapy patients but not in pre‐chemotherapy patients.

Several hypotheses have been proposed to explain the role of BMI in next‐generation anti‐androgen treatment. For example, biological studies showed androgen is converted to estrogen in adipose tissue^19^ and circulating estradiol has been proven to improve the survival. However, both cohorts showed no difference in baseline testosterone levels in the different BMI groups. Considering previous treatment may influence patients’ response to subsequent treatment, we then analyzed rPFS stratified by BMI in the pre‐ and post‐chemotherapy groups, but no significant differences in response were identified in either cohort (date not shown). Another reasonable speculation was that the low BMI group may have included more patients with cancer‐ or chemotherapy‐related cachexia than the high BMI group. Therefore, ALB, which is a readily detectable biomarker of cachexia,^20^, ^21^ was combined with BMI to evaluate its association with OS. As expected, in COU‐AA‐301, patients with normal BMI and low ALB had the worse OS while high BMI and normal ALB patients had the best prognosis. It is noteworthy that the outcome of patients with high BMI but low ALB was even worse than low BMI but normal ALB patients. These results were confirmed in COU‐AA‐302. We hypothesized that weight gain caused by androgen deprivation therapy or treatment‐related edema may lead to increased BMI, which conceals the potential cachexia status in patients. ALB levels reflecting the nutritional status of patients could better identify potential cachexia patients. Similar results were found in a study of the obesity paradox in clear cell renal cell carcinoma, which showed obesity patients with low ALB had a higher cumulative incidence of cancer‐specific death than normal weight patients with normal ALB.^22^ Taken together, our results suggest combining BMI and ALB could better predict the survival in mCRPC patients treated with abiraterone. High BMI with low ALB patients may represent a large but under‐recognized entity with a poor outcome that warrants further research.

The strength of our study was the large and independent cohorts that included patients with different treatment histories. Consistent results were found in both pre‐ and post‐chemotherapy patients, confirming the prognostic value of combined BMI and ALB. However, limitations in this study should be noted, including the selection bias resulting from this being a post hoc analysis and the absence of validation cohort because of the difference in inclusion. The two cohorts in this study were from clinical phase III trials and included patients that had a relatively better health status according to the inclusion and exclusion criteria. Combined BMI and ALB still predicted the prognosis in mCRPC patients and high BMI but low ALB patients were also identified as a group with under‐recognized hidden cachexia. Real‐world studies are likely to encounter a higher proportion of high BMI/low ALB patients and attenuate the effect size. Second, although we observed a strong association between combined BMI and ALB and poor outcome, a causal link cannot be concluded based on the current analysis. Therefore, certain confounders such as smoking and diet may explain the observed association, and a highly metabolic and aggressive tumor may account for the observed cachexia status and cause a reverse association. Therefore, a prospective clinical study focusing on nutritional status in mCRPC is required before employing such a potential intervention in a high‐risk population. Third, potential confounders such as diet and socioeconomic status cannot be adjusted in this study. A prospective clinical trial might address this problem in the future.

## 7 CONCLUSION

In conclusion, combining BMI with ALB better predicts the outcome of mCRPC patients. High BMI/low ALB mCRPC patients may represent a poorly understood group of cachexia patients with worse outcome. Early nutritional intervention for high BMI/low ALB patients should be investigated to optimize their survival.

## CONFLICT OF INTEREST

The authors declare no potential conflict of interest.

## AUTHOR CONTRIBUTIONS

Jian Pan: Data curation and formal analysis. Jun Wang: Data curation, formal analysis, visualization, and writing‐original draft. Yu Wei: Investigation and resources. Tingwei Zhang: Investigation and resources. Shen Zhang: Methodology and validation. Yao Zhu: Conceptualization, funding acquisition, supervision, and writing‐review. Dingwei Ye: Conceptualization, funding acquisition, supervision, resources, and writing‐review.

## Supporting information

Table S1

## Data Availability

All data included in this study are available upon request by contact with the corresponding author.
