# Supplementary material for: Combination of body mass index and albumin predicts the survival in metastatic castration‐resistant prostate cancer patients treated with abiraterone: A post hoc analysis of two randomized trials
Source: Cancer Med. 2021 Aug 20;10(19):6697–704. doi: 10.1002/cam4.4205 (PMC8495267; doi:10.1002/cam4.4205)
Supplement: Supplementary file 1 — Table S1 [file CAM4-10-6697-s001.docx]

**Supplement Table 1:** **Baseline characteristics of patients in the COU-AA-301 and COU-AA-302 cohort**

|  | COU-AA-301 (n=1172) | COU-AA-302 (n=1033) |
| --- | --- | --- |
| BMI, kg/m^2^, N (%) |  |  |
| <25 | 363 (31%) | 198 (19%) |
| ≥25 | 809 (69%) | 844 (81%) |
| ECOG, N (%) |  |  |
| 0 | 394 (34%) | 767(74%) |
| 1 | 626 (53%) | 253(24%) |
| 2 | 124 (11%) | 1(0%) |
| BPI-SF, N (%) |  |  |
| 0 | 245 (21%) | 515(50%) |
| 1 | 259 (22%) | 148 (14%) |
| 2 | 299 (26%) | 166 (16%) |
| 3 | 247 (21%) | 167 (16%) |
| 4 | 70 (6%) | 3 (0.29%) |
| Previous chemotherapy regimens, N (%) |  |  |
| 1 | 799 (68%) | - |
| 2 | 344 (29%) | - |
| Progression category, N (%) |  |  |
| PSA only | 343 (29%) | - |
| radiographic | 800 (68%) | - |
| Median PSA, ng/ml (IQR) | 127 (38-388) | 37 (14-99) |
| Median Hb, g/dl (IQR) | 11.9 (10.8-12.9) | 13.2 (12.4-13.9) |
| Median LDH, IU/l (IQR) | 228 (187-316) | 186 (162-215) |
| Median ALP, IU/l (IQR) | 127 (80-261) | 90 (70-129) |
| Median ALB, g/dl (IQR) | 4.1 (3.9-4.4) | 4 (3.8-4.2) |

BMI = body mass index; AAP = abiraterone acetate + prednisone; PP = placebo + prednisone; ECOG = Eastern Cooperative Oncology Group; BPI-SF = Brief Pain Inventory-Short Form; PSA = prostate-specific antigen; Hb = hemoglobin; LDH = lactate dehydrogenase; ALP = alkaline phosphatase; ALB = albumin; IQR = interquartile range.
